# Imaging features of hemangioma in long tubular bones

**DOI:** 10.1186/s12891-020-03882-2

**Published:** 2021-01-06

**Authors:** Lei Cao, Jin-Xu Wen, Shu-Man Han, Hui-Zhao Wu, Zhi-Gang Peng, Bao-Hai Yu, Zhi-Wei Zhong, Tao Sun, Wen-Juan Wu, Bu-Lang Gao

**Affiliations:** Department of Radiology the Third Hospital, Hebei Medical University, 139 Ziqiang Road, 050051 Shijiazhuang, Hebei Province China

**Keywords:** Hamangioma, Long bone, Plain radiography, Computed tomography, Magnetic resonance imaging.

## Abstract

**Background:**

To investigate the imaging features of hemangiomas in long tabular bones for better diagnosis.

**Methods:**

Twenty-four patients with long bone hemangiomas confirmed by pathology were enrolled. Nineteen patients had plain radiography, fourteen patients had computed tomography (CT) and eleven had magnetic resonance imaging (MRI). The hemangioma was divided into medullary [13], periosteal [6] and intracortical type [5].

**Results:**

Among 19 patients with plain radiography, eleven patients were medullary, three periosteal, and five intracortical. In the medullary type, the lesion was primarily osteolytic, including five cases with irregular and unclear rims and one lesion having osteosclerotic and unclear rims. In three patients with the periosteal type, the lesion had clear rims with involvement of the cortical bone in the form of bone defect, including two cases with local thickened bone periosteum and one case having expansile periosteum. Five intracortical hemangiomas had intracortical osteolytic lesions with clear margins. Among 14 patients with CT imaging, 8 cases were medullary, three periosteal, and three intracortical. Among 8 medullary hemangiomas, one had ground glass opacity, and seven had osteolytic, expansile lesions like soft tissue density with no calcification. In three periosteal cases, the lesion was osteolytic with thickened periosteum and narrowed medullary cavity. In three intracortical hemangiomas, the lesion was of even soft tissue density with no calcification. Among 11 patients with MRI imaging, seven were medullary, two periosteal, and two intracortical. Among 7 medullary lesions, six were of hypointense signal on T1WI and hyperintensesignal on T2 WI. In two periosteal cases, the periosteum was thickened, with one case being of equal signal, and the other having no signal. Two intracortical hemangiomas were both of slightly low signal on T1WI but hyperintense signal on T2WI.

**Conclusions:**

The long bone hemangiomas had characteristic cystic honeycomb-like presentations in plain radiograph. CT and MRI imagings are helpful for diagnosis of hemangiomas in long bone.

## Background

Osseous hemangioma accounts for 1% of all primary skeletal neoplasms with about 75% in the vertebra or skull and 15%-20% in the scapula, ribs, clavicle and pelvic bones [1–4]. Osseous hemangioma is frequently solitary but may be multiple. It is composed of thin-walled vessels or sinuses filled with blood interspersed among longitudinally oriented bony trabeculae, and lipid materials may be secondarily accumulated in this lesion [5]. The lesion is asymptomatic most of the time but may produce symptoms in cases of hematoma formation, epidural extension, vertebral body expansion, or pathological fracture [6–8]. This neoplasm usually occurs in the vertebra and craniofacial bones, and long tubular bones in four extremities have been infrequently involved [9–11]. Some doctors noted that even with vast experience with bone lesions, one can rarely encounters a primary hemangioma of the long tubular bones [2]. On plain radiography, vertebral hemangiomas take the typical corduroy appearance, presenting with thickened vertical trabecula of bone. On computed tomography (CT) images, a typical “polka dot” pattern is presented with small foci of sclerosis which stands for the thickened vertically oriented bony trabecula, and areas of low-density soft tissues or abnormal vessels and lipid are intervening between the vertically-oriented trabecula [5]. Hemangiomas have typical high signal intensity on both T1WI and T2WI of magnetic resonance imaging (MRI) because of abundant adipocytes, blood vessels and interstitial edema [5]. Radiological characteristics of this tumor are so typical in the spine and skull that radiologists can easily differentiate it from other forms of bone tumors. Contrary to the hemangioma in the skull and spine with typical clinical presentations, correct diagnosis of this neoplasm may be very challenging when it occurs in the long tubular bones or demonstrates atypical imaging features. Because of its rarity, hemangioma of the peripheral long bones may pose diagnostic difficulties and cause unnecessary redundant examinations in radiology and laboratory. We presented a series of patients with hemangiomas in the long tubular bones proved by histopathology and analyzed the imaging characteristics so as to increase the diagnostic accuracy for this tumor.

## Methods

This study was approved by our hospital ethics committee with all the patients given their signed informed consent. Between 2000 and 2019, twenty-four patients with hemangiomas in the long tubular bones were confirmed by histopathology including 13 males and 11 females with an age range of 11–67 years (median 35). The presentation symptoms and signs were pain in 20 cases and local masses in six patients with a history of one month to 10 years. Plain radiography was performed in 19 cases, CT in 14, and MRI in 11.

All the images were analyzed by two senior imaging physicians with 15 years of experience, and when in disagreement, a third radiologist with 20 years of experience was involved to reach a consensus. The lesions were divided into medullary (the lesion was located in the medulla or at the cancellous bone), periosteal (the lesion was located at the surface of bone), and intracortical (the lesion was inside the cortex). 

## Results

### Types of lesion

The long bone hemangioma was at the femur in 9 patients, tibia in 7, fibula in four, humerus in 3, and radius in the remaining one, including medullary hemangioma in 13 cases (54.2%), periosteal in 6 (25%) and intracortical in the remaining 5 (20.8%).

### Plain radiography

**Fig. 1:**
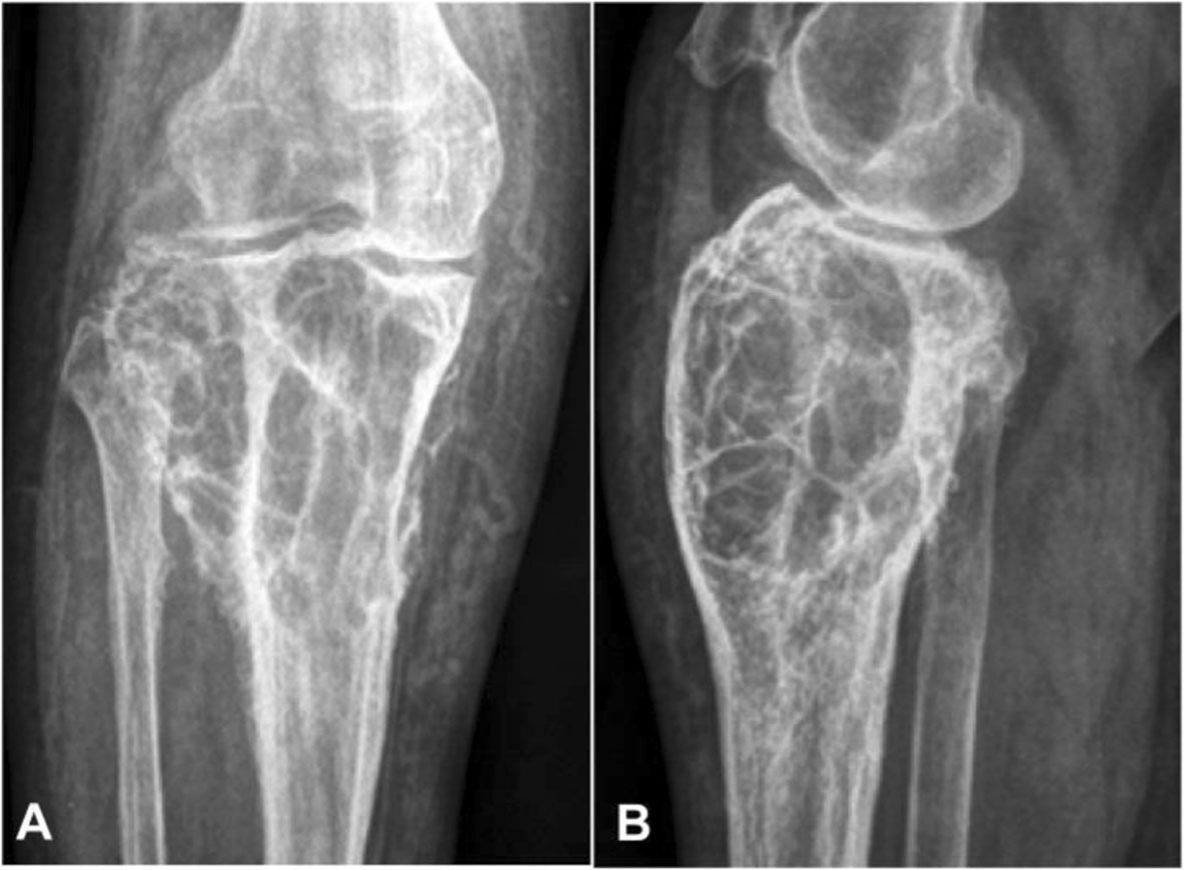
Medullary hemangioma in the proximal end of tibia. Plain radiograph of the anteroposterior (**a**) and lateral view (**b**) of the proximal tibia and fibula showed an eccentric, expansile, osteolytic lesion at the proximal end of the tibia, with coarse bone ridges inside the lesion like a honeycomb. The cortical bone was thinned, periosteal reaction was presented, and tortuous vascular structures were seen in the interior soft tissue

**Fig. 2:**
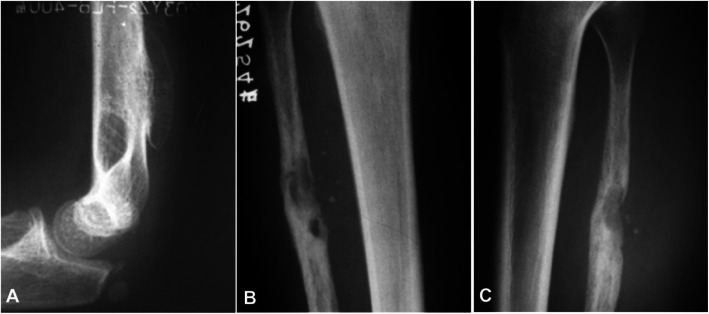
Periosteal hemangioma. **a**. Plain radiograph of the lateral view of distal humerus showed an expansile lesion at the lateral humerus with radial bone crest perpendicular to the bone cortex inside the lesion. Coarse ridges were also seen inside the lesion. **b** & **c**. Pain radiograph of the anteroposterior view of the middle and upper fibula revealed that the middle and upper segments of the right fibula were enlarged with irregular osteosclerosis and low-density lesions inside. Local bone cortex was depressed, forming a pressure trace, and punctate calcification was demonstrated inside the adjacent soft tissue

**Fig. 3:**
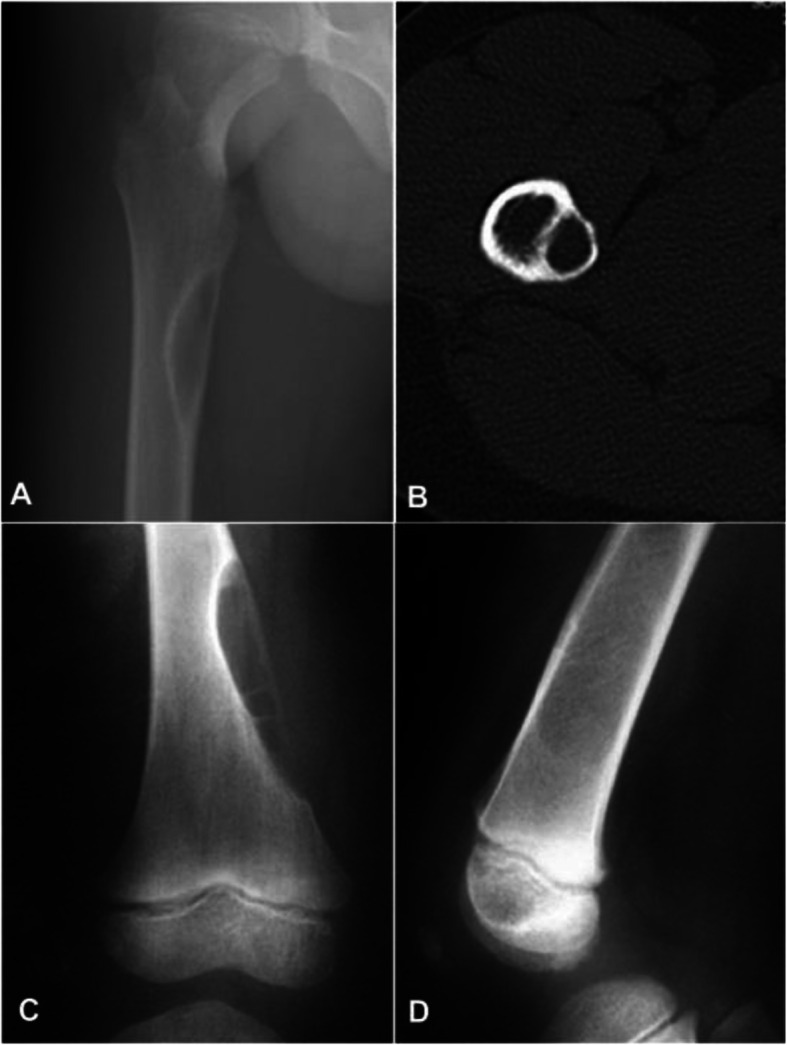
Imaging of intracortical hemangioma. **a** & **b**. Plain radiograph of the anteroposterior view of the proximal femur (**a**) and computed tomography imaging in transverse view (**b**) demonstrated an intracortical hemangioma lesion below the right femoral trochanter. The lesion was fusiform with the long axis parallel to the long axis of the femur and was expansile and osteolytic with osteodermic sclerosis at the edge. **c** & **d**. Plain radiographs of the anteroposterior (**c**) and lateral view (**d**) of the distal femur revealed an osteolytic bone defect with septa inside the lesion at the distal interior end of the left femur. The medullary side of the lesion was osteoslerotic with discontinuation of the interior bone cortex

Nineteen patients had plain radiography with 11 patients presenting as medullary, three as periosteal and the rest five as intracortical hemangiomas. In medullary hemangioma, the lesion was lytic accompanied with coarse trabecula, with a beehive-like appearance in five patients, a sclerotic rim in three patients (Fig. 1), irregular osseous sclerosis in two, and central osteolysis in one. Among the 11 patients with medullary hemangioma, irregular ill-defined rims were found in five cases, irregular periosteal hyperplasia in three, a soft tissue mass in one, and strip-like calcification in one. Among three patients with periosteal hemangioma, the lesion was in the fibula in two and humerus in the rest one, with periosteal hyperplasia in two cases and eccentrically expansile lesion in one fibular case with a lot of pathological ossification forming a bone shell (Fig. 2a). One case had calcification in the surrounding soft tissue (Fig. 2b&c). Among five patients with intracorticl hemangioma, the lesion was lytic inside the cortex with the long axis of the lesion parallel to that of the long bone with clear rims but no periosteal reaction (Fig. 3a&b). In four cases, the lesion was of lytic expansile appearance inside the thickened cortex (Fig. 3c&d).

### CT images

**Fig. 4:**
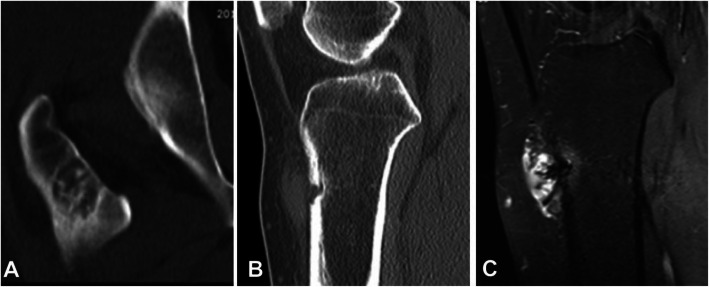
Medullary and intracortical hemangiomas. **a**. Coronal reconstruction of computed tomography (CT) imaging showed that the medullary hemangioma lesion was located inside the medullary cavity in the intertrochanteric femur with bone septations and osteosclerotic rims. **b** & **c**. Sagittal reconstruction of CT imaging (**b**) and axial T2WI magnetic resonance imaging (**c**) showed local wedge bone destruction with tortuous vessels in adjacent soft tissue. Enhancement scanning (**b**) displayed uneven enhancement in the vessels and adjacent medullary cavity

Fourteen patients had CT scanning with a medullary type in eight patients, periosteal in three, and intracortical in the remaining three. Among eight patients with medullary hemangioma, a ground-glass appearance was in one while a lytic expansile lesion without apparent calcification was demonstrated in the other seven cases including four cases having a sclerotic rim with varying thickness, one with incomplete sclerotic rim, and two without sclerotic rim. Among eight medullary lesions, four cases had bone septa inside the lesion like a beehive (Fig. 4a), one had sieve-hole-like cortex like metastatic lesions, two cases had radial periosteal reaction perpendicular to the cortex, and the remaining one had irregular periosteum. Among three cases with a periosteal type lesion, the lesion was lytic and surrounded by thickened periosteum with stenotic marrow cavity. The cortex was thickened evenly in two cases but unevenly in the other one (Fig. 4a). Among three cases with intracortical type, the lesion was like soft tissues in even density with local expansile thickened cortex in two cases, and discontinued cortex and enhanced tortuous vessels in the other one (Fig. 4b). Punctiform calcification was present in the soft tissue in two of the 14 cases with CT scanning.

### MRI images

**Fig. 5:**
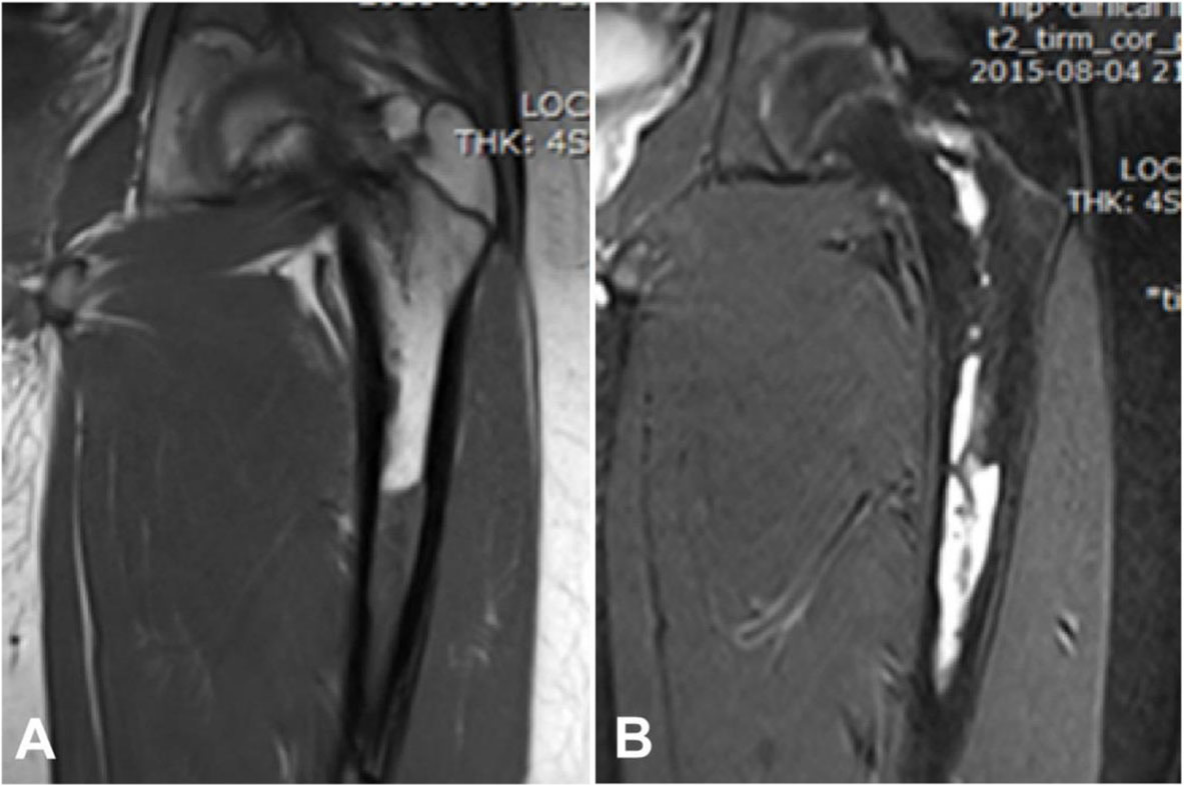
Medullary hemangioma. Coronal T1WI (**a**) and T2WI (**b**) showed that the medullary hemangioma lesion was located inside the medullary cavity, extending downwards to the upper and middle segments of the femur, in hypointense signal on T1WI and hyperintense signal on T2WI. The lesion was like expanded vessels with clear margins

Eleven patients had MRI scan including seven cases of the medullary type, two of the periosteal type, and two of the intracortical type. Among the seven cases with the medullary type, four cases had well-defined lesions of hypointense signal on T1WI but hyperintense signal on T2WI compared with normal cancellous bone, of which two cases had honeycomb-like septations. In one case, the lesion broke through the bone cortex and had uneven T1WI slight hypointense signal and high signal on T2WI, with the adjacent medullary cavity being of hypointense signal on in T1WI and hyperintense signal on T2WI compared with normal medullary cavity. In one case, the lesion was oval at the medulla of the neck of femur, which had slight hypointense signal on T1WI but hyperintense signal on T2WI, with honeycomb-like septations. The abnormal signal extended downwards to the upper and middle segment of the femur, with clear margins and uneven thinned cortex at the internal side (Fig. 5). In one case, the lesion had equal or low signal compared with the normal medullary cavity on both T1WI and T2WI, with ill-defined margins and periosteal reaction. In two cases with periosteal type, the periosteum was thickened with one case having isointense signal (compared to normal medullary cavity) and one with no signal, and within the soft tissue in both cases, slightly hypointense signal on T1WI and hyperintense signal on T2WI were demonstrated. In two cases with the intracortical type, the lesion was slightly hypointense signal on T1WI but hyperintense signal on T2WI including one case with expansile growth and clear rims and the other of ill-defined rimes with non-uniform signal of the soft tissue mass and tortuous enhanced vessels (Fig. 4c).

## Discussion

Hemangioma is a rare bone lesion with a preponderance in females, with most patients being young adults and female patients younger than their male counterparts [2]. Contrary to vertebral or skull hemangiomas which are mostly asymptomatic, extremity hamgiomas are symptomatic in most patients, with local swelling or a soft tissue mass in 77% [2, 4, 12]. Pathological fractures are uncommon and may be the initial clinical presentation in less than 10% of patients. In our study, the symptoms included local pain in 20 cases and a local mass and swelling in six cases. The duration of symptoms is quite variable from a few days to many years, and in our case series, the symptom duration ranged from one month to 10 years.

The lower extremities are the predominant location for skeletal hemangioma (73%), with the tibia and femur being the commonest site, similar to our case series. It has been reported that most hemangiomas were meduallary (55%) followed by periosteal (33%) and intracortical (12%) [2]. This was also consistent with our report with the medullary hemangioma in 13 cases (54.2%), periosteal in six (25%) and intracortical in the remaining five (20.8%). Most skeleton hemangiomas (80%) take place in the diaphysis or metadiaphysis, with the metaphysis being solely involved in only 10% [2]. In histopathology, bone hemangioma can be divided into three types: cavernous, capillary and venous, with the cavernous hemangioma being the commonest type in extremity long bones, accounting for greater than one-half of all cases. Venous hemangiomas are composed of blood vessels with thickened walls of smooth muscles without arterial components and have been rarely reported in the long bones. Pure capillary hemangiomas are also uncommon in long bones, and only about 10% are capillary hemangioma which all occur in long bones. Bone hemangioma is developed from embryonal hemangioblasts and is characterized by vascular neoplastic malformation, thin-walled capillary, vascular hyperplasia, and bloody sinuses.

Radiological presentations of hemangiomas in the long bones are highly variable and non-specific and are difficult for a correct diagnosis [4] because diverse matrix found in the bone hemangioma like vessels, fat, fibrous tissue, smooth muscles, and clotted blood may all account for the variable radiological presentations [13, 14]. The medullary type mainly occurred at the diaphysis or metaphysis with few involvement of the epiphysis. This type of lesion usually presents as an osteolytic lesion with internal trabecula in the appearance of honeycomb or soap bubble, resulting from expansile proliferation of engorged vessels and thickened remodeled bone trabecula. Spiculated periosteal reaction may be presented, and medullary hemangioma may present as completely lucent on radiolology [15]. In our opinion, a lytic lesion accompanied with coarse trabecula or a sclerotic rim with varying thickness in the long bones may indicate hemangioma.

CT appearance of hemangioma in long bones correspond to that on radiographs as a lytic lesion with internal trabecula [14], and in some patients, the thickened vertical trabecular may have a ‘polka dot’ appearance as in the spinal variety [13, 14]. On MRI, the long bone hemangioma may demonstrate low, intermediate or high signal intensity on T1WI. Hyperintense signal on T1WI may be caused by the fat content within the lesion, whereas hyperintense signal on T2WI may be due to the fluid content of the tumor vessels [4]. Characteristically hypointense signal of the internal trabecular may be shown on the T1WI and T2WI in the long bone hemangioma [13]. Marked to minimal or no enhancement may be demonstrated in the long bone hemangioma on post-contrast FST1 WI images [12, 15]. Cortical involvement is identified by elevated signal within the normally dark cortex. Coarse trabecula is generally not visible with standard MRI imaging but may be seen as low-signal, irregular lines within the lesion or as hypointense signal dots within a hyperintense signal background on high-resolution MRI. The MRI role lies in defining the nature and scope of the lesion and differentiating other easily confused diseases. Razek et al [16] had investigated the diagnostic accuracy of diffusion tensor imaging in differentiation of malignant from benign compressed vertebra. They believed that diffusion weighted MR imaging and derived apparent diffusion coefficient maps may help to define the nature of the lesions and provide qualitative and quantitative information on the tissues studied. The non-invasive diffusion tensor imaging technology can provide accurate imaging parameters to distinguish malignant tumors from benign compressed vertebra. Their study provides some enlightening ideas for accurate differentiation of benign and malignant bone tumors and for prospective diffusion tensor imaging research of osteo-hemangiomas in the future [16]. After investigating MRI findings of intraosseous hemangioma in long tubular bones of 15 cases, Zhou et al. [17] found that all these intraosseous hemangioma lesions had high intensity on T2WI but intermediate signal intensity on T1WI, with two lesions confusing on radiology but being clearly confirmed on MRI. In our study, some cases presented with low signal intensity on T1WI, whereas others had the same or lower signal intensity on both T1WI and T2WI compared with normal medullary cavity, unclear boundary, and periosteal reaction. These new findings help to improve the diagnostic accuracy of hemangiomas in long tubular bones. Compared with previous studies [12, 17, 18], our study had a greater number of cases, complete imaging data, diverse imaging manifestations, and multiple long tubular bones, with some manifestations of hemangioma being similar to those of malignant tumors (such as lymphoma). By describing the common characteristic presentations (coarse bone trabecular bone) and uncommon non-characteristic manifestations, it is helpful for correct differential diagnosis.

Intramedullary hemangiomas need to be differentiated from other chondrogenic or dilative dissolved tumors, such as giant cell tumor, aneurysmal bone cyst, or fibrous dysplasia. In these cases, reticular or honeycomb lesions can also be seen, with thick internal trabecula. Sometimes, it is difficult to make a correct diagnosis only by X-ray radiography and CT. Intra-hemangioma cyst is easy to be misdiagnosed as bone cyst or fibrous dysplasia, which needs MRI to identify. In periosteal hemangioma, thickening of cortical bone indicates that the lesion has involved the periosteum. With enrichment of periosteal blood supply, osteoblasts become more active, leading to periosteal hyperplasia to gradually manifest as cortical bone thickening. This type of hemangioma needs to be distinguished from osseous structural changes, because soft tissue hemangioma can also readily invade adjacent bones, resulting in cortical thickening. MRI is helpful to determine the location and scope of the lesion, which is conducive to differential diagnosis. Cortical hemangioma is easy to be confused with osteoid osteoma. MRI is superior to X-ray and CT in determining the location, boundarys and extent of lesions [12, 17]. When linear ossification and extraosseous extension are present in dilative dissolved diseases, these atypical features may increase the suspicion for malignant tumors such as lymphoma, metastasis, osteomyelitis, or chondrosarcoma [13], and further advanced imaging examination and even pathological biopsy are needed for a correct diagnosis. Both benign and malignant soft tissue tumors may affect adjacent bones. Soft tissue hemangioma invading adjacent bones will cause periosteal thickening and bone cortical changes, similar to some signs of bone hemangioma, whereas malignant soft tissue tumors can also have similar presentations, difficult for differentiation. After studying soft tissue tumors of the extremities with diffusion echo-planar MRI, Razek et al. [19] found that the mean apparent diffusion coefficient value of malignant soft tissue tumors was significantly (P < 0.001) lower than that of benign masses (1.02 ± 0.03 × 10^− 3^mm^2^/s vs. 1.54 ± 0.03 × 10^− 3^mm^2^/s). Diffusion-weighted echo-planar MRI is a promising non-invasive modality which may be helpful for differentiating malignant from benign soft tissue tumors and for grading malignancy.

In our study, the plain radiography, CT, and MRI images of hemangioma in long tubular bones were investigated. The advantages of plain radiography are convenient and fast, with low medical cost but high spatial resolution, which can be used to evaluate the location and scope of lesions, but the disadvantage is its inability to clearly show the lesion details and density differences. Thus, plain radiography should be used as the first level examination method, which is suitable for small lesions with slight bone changes and for follow-up of these patients. CT provides the clearest description of cortical destruction and is an excellent imaging method for characterizing tumor matrix, exact location, expansion, and bone changes. However, the display of soft tissue on CT imaging is weaker than that of MRI, and it can be used as a secondary examination method for the diagnosis of large lesions and severe bone destruction. MRI can be used to observe periosteal reaction, bone marrow edema, and soft tissue edema. Moreover, MRI can be used to quantitatively evaluate the composition of lesions and the characteristics of blood flow signals, but it is expensive and may serve as the third level examination method for diagnosis. Understanding the advantages and disadvantages of each imaging technique is helpful to establish the correct imaging examination procedure for a correct diagnosis.

For correct diagnosis of hemangioma in long tubular bones, plain radiography should be performed firstly when local symptoms in the extremities appear, and small benign lesions can be diagnosed and followed up for close monitoring. For larger expansile lesions with unclear margins, CT scanning should be conducted for differentiation and for further decision of follow-up observation or biopsy. If CT imaging cannot determine the malignancy or benign nature of a lesion, or when surgery is needed, MRI scanning should be performed to determine the nature of the lesion and to provide detailed information of the lesion.

## Conclusions

In conclusion, long bone hemangiomas are rare and may, although not-life threatening, pose a challenging task in diagnosis because of variable radiological appearances, and classical radiological presentations including coarse trabecular bone pattern or soap-bubble appearances may suggest this disease.

## Data Availability

All data and materials are available from the corresponding author on reasonable requirements.
